# Sigma-1 receptor deficiency reduces MPTP-induced parkinsonism and death of dopaminergic neurons

**DOI:** 10.1038/cddis.2015.194

**Published:** 2015-07-23

**Authors:** J Hong, S Sha, L Zhou, C Wang, J Yin, L Chen

**Affiliations:** 1 State Key Lab of Reproductive Medicine, Nanjing Medical University, Nanjing, China; 2 Department of Physiology, Nanjing Medical University, Nanjing, China

## Abstract

Sigma-1 receptor (*σ*_1_R) has been reported to be decreased in nigrostriatal motor system of Parkinson's disease patients. Using heterozygous and homozygous *σ*_1_R knockout (*σ*_1_R^+/−^ and *σ*_1_R^−/−^) mice, we investigated the influence of *σ*_1_R deficiency on 1-methyl-4-phenyl-1,2,3,6-tetrahydropyridine (MPTP)-impaired nigrostriatal motor system. The injection of MPTP for 5 weeks in wild-type mice (MPTP-WT mice), but not in *σ*_1_R^+/−^ or *σ*_1_R^−/−^ mice (MPTP-*σ*_1_R^+/−^ or MPTP-*σ*_1_R^−/−^ mice), caused motor deficits and ~40% death of dopaminergic neurons in substantia nigra pars compacta with an elevation of *N*-methyl-d-aspartate receptor (NMDAr) NR2B phosphorylation. The *σ*_1_R antagonist NE100 or the NR2B inhibitor Ro25-6981 could alleviate the motor deficits and the death of dopaminergic neurons in MPTP-WT mice. By contrast, MPTP-*σ*_1_R^+/−^ mice treated with the *σ*_1_R agonist PRE084 or MPTP-*σ*_1_R^−/−^ mice treated with the NMDAr agonist NMDA appeared to have similar motor deficits and loss of dopaminergic neurons as MPTP-WT mice. The pharmacological or genetic inactivation of *σ*_1_R suppressed the expression of dopamine transporter (DAT) in substantia nigra, which was corrected by NMDA. The activation of *σ*_1_R by PRE084 enhanced the DAT expression in WT mice or *σ*_1_R^+/−^ mice. By contrast, the level of vesicular monoamine transporter 2 (VMAT2) in *σ*_1_R^+/−^ mice or *σ*_1_R^−/−^ mice had no difference from WT mice. Interestingly, MPTP-WT mice showed the reduction in the levels of DAT and VMAT2, but MPTP-*σ*_1_R^−/−^ mice did not. The inactivation of *σ*_1_R by NE100 could prevent the reduction of VMAT2 in MPTP-WT mice. In addition, the activation of microglia cells in substantia nigra was equally enhanced in MPTP-WT mice and MPTP-*σ*_1_R^−/−^ mice. The number of activated astrocytes in MPTP-*σ*_1_R^−/−^ mice was less than that in MPTP-WT mice. The findings indicate that the *σ*_1_R deficiency through suppressing NMDAr function and DAT expression can reduce MPTP-induced death of dopaminergic neurons and parkinsonism.

Parkinson's disease (PD) is a neurodegenerative disorder characterized by motor symptoms, including bradykinesia and tremor, and a progressive loss of dopaminergic neurons in substantia nigra pars compacta (SNpc).^1, 2^ Sigma-1 receptor (*σ*_1_R), previously named the opioid receptor sigma-1, is found primarily in motoneurons localized in the brainstem and spinal cord.^3^ The *σ*_1_R is expressed in dopaminergic neurons and astrocytes.^4^ The *σ*_1_R agonist PRE084 has been reported to exert neurorestorative effects on 6-hydroxydopamine (6-OHDA)-induced parkinsonism.^4^ Using positron emission tomography, the *σ*_1_R-binding sites are found to be reduced in the brains of early-phase PD patients.^5^ However, the influence of *σ*_1_R deficiency on the pathogenesis of PD has not yet been reported.

Dopamine toxicity is involved in the etiology of PD.^6^ The *σ*_1_R-binding sites on dopaminergic nerve terminals are involved in increasing dopamine release by enhancing *N*-methyl-d-aspartate receptors (NMDAr).^7^ The neurotoxin 1-methyl-4-phenyl-1,2,3,6-tetrahydropyridine (MPTP) metabolized to 1-methyl-4-phenylpyridinium in glial cells selectively impairs dopaminergic neurons in SNpc through disrupting respiratory enzymes and causing oxidative damage.^8^ The dopamine transporter (DAT), a high-affinity transmembrane protein, is responsible for dopamine reuptake from the synaptic cleft and the transportation of 1-methyl-4-phenylpyridinium into dopaminergic nerve terminals.^9^ The *σ*_1_R is co-expressed with DAT in dopaminergic neurons.^4^ Furthermore, the low density of DAT has been confirmed in the brains of PD patients.^5^

The activation of *σ*_1_R enhances the Ca^2+^ influx across NMDAr through increasing the phosphorylation of NR2B or the trafficking NMDAr to the plasma membrane.^10, 11^ The NMDAr NR2B inhibitor can attenuate MPTP- or 6-OHDA-induced parkinsonian symptoms and neurodegeneration.^12^ The *σ*_1_R deficiency has been demonstrated to reduce A*β*-induced neuronal cell death through suppressing NR2B phosphorylation.^13^ The inflammation is a predominant aspect of PD, manifested by glial activation with the expression of pro-inflammatory mediators.^14^ Sustained neuro-inflammation can exacerbate the degeneration of dopaminergic neurons.^15^ The blockade of *σ*_1_R has been reported to inhibit methamphetamine-induced astrogliosis.^16^ Moreover, the 6-OHDA-induced spontaneous rotations or decline of dopaminergic fibers in *σ*_1_R knockout mice seem to be less than those in wild-type (WT) mice.^4^ Paquette *et al.* reported that the blockade of *σ*_1_R could attenuate abnormal involuntary movements induced by 6-OHDA.^17^

In this study, we employed heterozygous and homozygous *σ*_1_R knockout (*σ*_1_R^+/−^ and *σ*_1_R^−/−^) mice to investigate the influence of *σ*_1_R deficiency on MPTP-induced parkinsonism and death of dopaminergic neurons, and the underlying molecular mechanisms. Using the experimental PD models of MPTP-treated *σ*_1_R^+/−^ mice and *σ*_1_R^−/−^ mice, the present study provides *in vivo* evidence that the *σ*_1_R deficiency through suppressing NMDAr function and DAT expression can attenuate MPTP-induced dopaminergic neurodegeneration and parkinsonism.

## Results

### *σ*_1_R deficiency reduces MPTP-induced motor deficits

**Figure 1:**
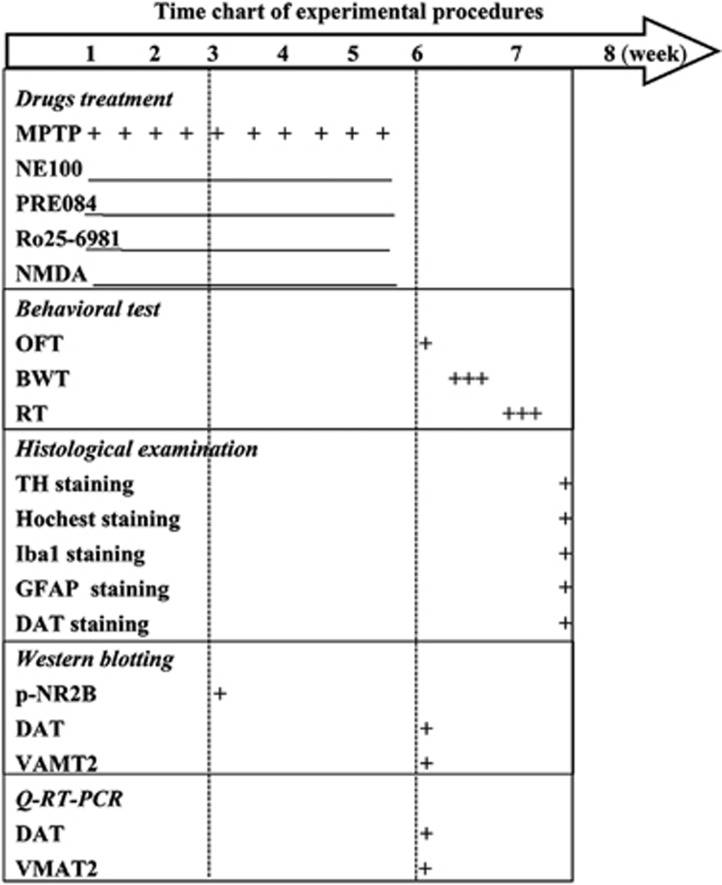
Time chart of experimental procedures. MPTP was injected for 10 times within 5 weeks. The *σ*_1_R antagonist NE100, *σ*_1_R agonist PRE084, NR2B inhibitor Ro25-6981 or NMDAr agonist NMDA was daily injected (i.p.) for 5 weeks. Open-field test (OFT), beam walking test (BWT) and rotarod test (RT) were performed on days 3–11 after last MPTP injection. Immunohistological staining of TH, Hoechst, Iba1 and GFAP was performed on day 12 after last MPTP injection

**Figure 2:**
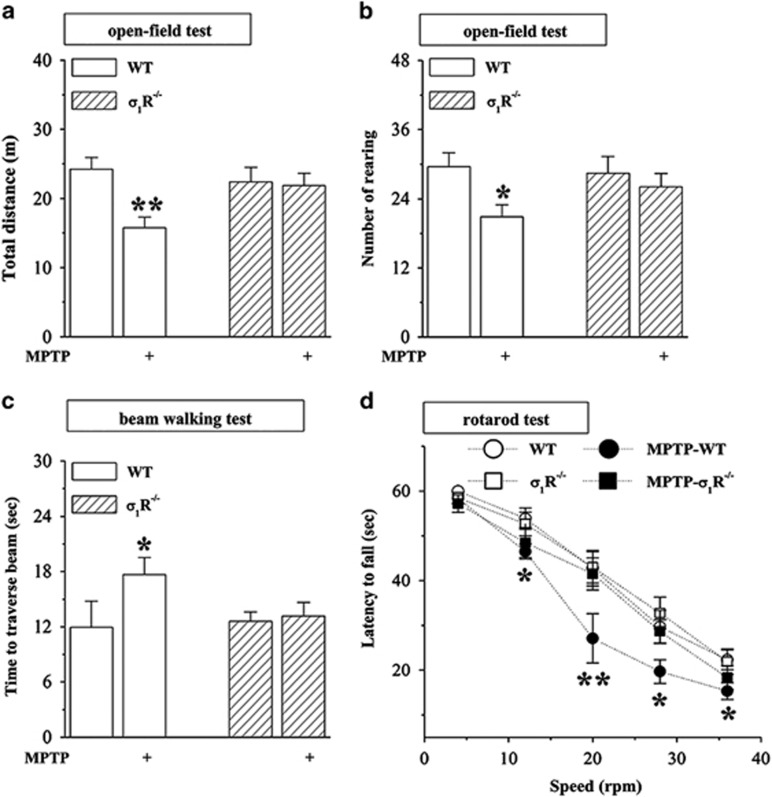
*σ*_1_R deficiency reduces MPTP-induced motor deficits. (**a–d**) Influence of *σ*_1_R deficiency on MPTP-induced motor deficits. Bar graphs show the traveled distance (m) and rearing number within 6 min in OFT (**a** and **b**), time (sec) to traverse the beam in BWT (**c**), latency (sec) to fall off the rotated rod in RT (**d**) in WT mice (WT) and *σ*_1_R^−/−^ mice (*σ*_1_R^−/−^) treated with MPTP injection. Two-way ANOVA, (**a**) MPTP: F_(1, 44)_=6.264, *P*=0.016; genotype: F_(1, 44)_=1.382, *P*=0.246; MPTP × genotype: F_(1, 44)_=4.843, *P*=0.033; **P<0.01 *versus* WT mice (Fisher's LSD); (**b**) MPTP: F_(1, 44)_=5.358, *P*=0.025; genotype: F_(1, 44)_=0.649, *P*=0.425; MPTP × genotype: F_(1, 44)_=3.119, *P*=0.084; *P<0.05 *versus* WT mice; (**c**) MPTP: F_(1, 44)_=4.509, *P*=0.039; genotype: F_(1, 44)_=1.652, *P*=0.205; MPTP × genotype: F_(1, 44)_=3.001, *P*=0.090; *P<0.05 *versus* WT mice. Repeated-measures ANOVA, (**d**) MPTP: F_(1, 44)_=19.473, *P*<0.001; genotype: F_(1, 44)_=8.767, *P*=0.005; MPTP × genotype: F_(1, 44)_=5.384, *P*=0.025; *P<0.05 and **P<0.01 *versus* WT mice (Fisher's LSD)

The locomotion ability and motor coordination were examined on days 3–11 after the last MPTP injection (Figure 1). In the open-field test (OFT), the traveled distance (Figure 2a) and the rearing number (Figure 2b) were not significantly different between WT and *σ*_1_R^−/−^ mice. In comparison with WT mice, MPTP-treated WT mice (MPTP-WT mice) showed a significant decrease in the traveled distance (*P*<0.01, *n*=12) and the rearing number (*P*<0.05, *n*=12), whereas MPTP-treated *σ*_1_R^−/−^ mice (MPTP-*σ*_1_R^−/−^ mice) did not (*P*>0.05, *n*=12).

In beam walking test (BWT), the walking time to traverse the beam in *σ*_1_R^−/−^ mice did not differ significantly from WT mice (*P*>0.05, *n*=12; Figure 2c). Notably, the prolongation of walking time to traverse the beam was observed in MPTP-WT mice compared with WT mice (*P*<0.05, *n*=12), but not in MPTP-*σ*_1_R^−/−^ mice (*P*>0.05, *n*=12).

The rotarod test (RT) is considered to be one of the most reliable tests to estimate motor coordination skills.^18^ The time (latency) that *σ*_1_R^−/−^ mice stayed on the rotated rod with speeds (4–36 r.p.m.) before falling off was not significantly different from WT mice (*P*>0.05, *n*=12). In comparison with WT mice, the latency on the rotated rod was markedly reduced in MPTP-WT mice (12, 28 and 36 r.p.m.: *P*<0.05; 20 r.p.m.: *P*<0.01, *n*=12; Figure 2d), but not in MPTP-*σ*_1_R^−/−^ mice (*P*>0.05, *n*=12).

### Blocked *σ*_1_R alleviates MPTP-induced motor deficits

**Figure 3:**
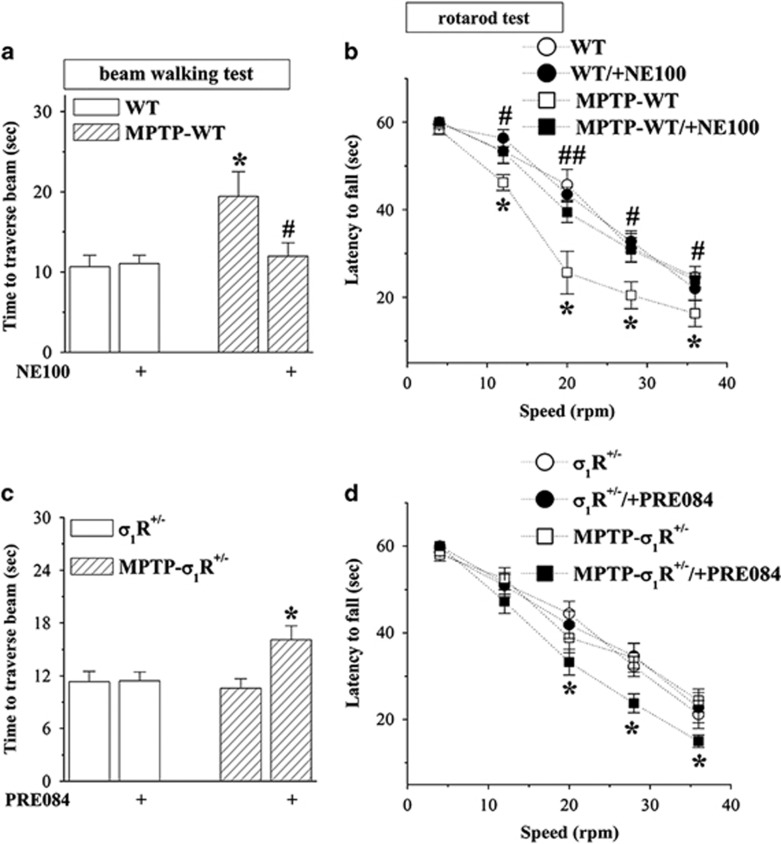
Blocked *σ*_1_R alleviates MPTP-induced motor deficits. (**a** and **b**) Effects of *σ*_1_R antagonist NE100 on MPTP-induced motor deficits. (**c** and **d**) Influence of *σ*_1_R agonist PRE084 on motor behaviors in *σ*_1_R^+/−^ mice and MPTP-*σ*_1_R^+/−^ mice. Bar graphs show time (s) to traverse the beam and latency (s) to fall off the rotated rod in WT mice and MPTP-WT mice treated with NE100, and *σ*_1_R^+/−^ mice and MPTP-*σ*_1_R^+/−^ mice treated with PRE084. Two-way ANOVA, (**a**) MPTP: F_(1, 44)_=8.215, *P*=0.006; NE100: F_(1, 44)_=1.993, *P*=0.165; MPTP × NE100: F_(1, 44)_=3.543, *P*=0.066; *P<0.05 *versus* WT mice; ^#^*P*<0.05 *versus* MPTP-WT mice (Fisher's LSD). Repeated-measures ANOVA, (**b**) MPTP: F_(1, 44)_=16.591, *P*<0.001; NE100: F_(1, 44)_=10.583, *P*=0.002; MPTP × NE100: F_(1, 44)_=12.185, *P*=0.001; *P<0.05 *versus* WT mice; ^#^*P*<0.05 and ^##^*P*<0.01 *versus* MPTP-WT mice (Fisher's LSD). Two-way ANOVA, (**c**) MPTP: F_(1, 44)_=2.514, *P*=0.120; PRE084: F_(1, 44)_=5.109, *P*=0.029; MPTP × PRE084: F_(1, 44)_=4.809, *P*=0.034; *P<0.05 *versus* MPTP-*σ*_1_R^+/−^ mice. Repeated-measures ANOVA, (**d**) MPTP: F_(1, 44)_=8.236, *P*=0.006; PRE084: F_(1, 44)_=4.954, *P*=0.031; MPTP × PRE084: F_(1, 44)_=5.307, *P*=0.026; *P<0.05 *versus* MPTP-*σ*_1_R^+/−^ mice (Fisher's LSD)

To confirm the involvement of *σ*_1_R in MPTP-induced motor deficits, MPTP-WT mice were treated with the *σ*_1_R antagonist NE100 (1 mg/kg), and MPTP-treated *σ*_1_R^+/−^ mice (MPTP-*σ*_1_R^+/−^ mice) were treated with the *σ*_1_R agonist PRE084 (1 mg/kg) for 5 weeks (Figure 1). The administration of NE100 could correct the prolongation of walking time on the challenging beam (*P*<0.05, *n*=12; Figure 3a) and the reduction of latency on the rotated rod (12, 28 and 36 r.p.m.: *P*<0.05; 20 r.p.m.: *P*<0.01, *n*=12; Figure 3b) in MPTP-WT mice, but it failed to affect these motor behaviors in WT mice (*P*>0.05, *n*=12). In comparison with *σ*_1_R^+/−^ mice, MPTP-*σ*_1_R^+/−^ mice did not show the changes in the walking time on the challenging beam (*P*>0.05, *n*=12; Figure 3c) and the latency on the rotated rod (*P*>0.05, *n*=12; Figure 3d). The treatment with PRE084 in MPTP-*σ*_1_R^+/−^ mice caused the prolongation of walking time on the challenging beam (*P*<0.05, *n*=12) and the reduction of latency on the rotated rod (20–36 r.p.m.: *P*<0.05, *n*=12), whereas it did not affect these motor behaviors in *σ*_1_R^+/−^ mice (*P*>0.05, *n*=12).

### *σ*_1_R deficiency reduces MPTP-induced death of dopaminergic neurons

**Figure 4:**
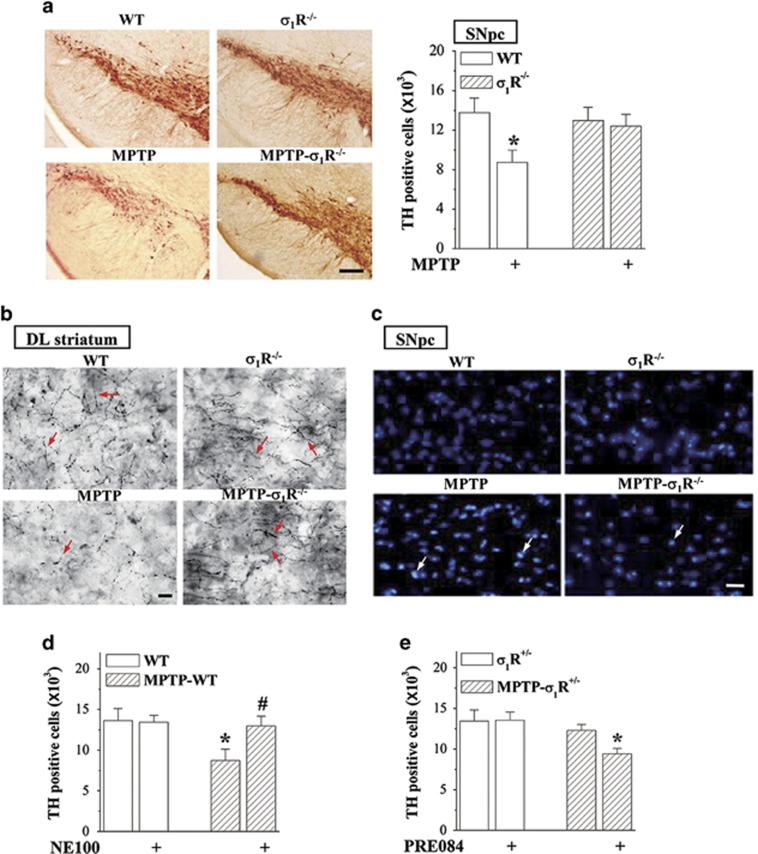
*σ*_1_R deficiency reduces the MPTP-induced death of dopaminergic neurons. (**a**) Stereological counts of TH-positive cells throughout SNpc in WT mice and *σ*_1_R^−/−^ mice treated with MPTP injection. Representative photomicrographs of TH staining. Scale bar=200 *μ*m. (**b**) Representative photomicrographs of TH-positive fibers (red arrows) in dorsolateral (DL) striatum. Scale bar=20 *μ*m. (**c**) Representative photomicrographs of Hoechst-positive cells (white arrows) in SNpc. Scale bars=50 *μ*m. (**d**) Effects of *σ*_1_R antagonist NE100 on MPTP-induced death of dopaminergic neurons. (**e**) Influence of *σ*_1_R agonist PRE084 on dopaminergic neurons in *σ*_1_R^+/−^ mice and MPTP-*σ*_1_R^+/−^ mice. Bar graphs show stereological counts of TH-positive cells. Two-way ANOVA, (**a**) MPTP: F_(1, 28)_=4.834, *P*=0.036; genotype: F_(1, 28)_=0.930, *P*=0.343; MPTP × genotype: F_(1, 28)_=2.455, *P*=0.128; *P<0.05 *versus* WT mice (Fisher's LSD); (**d**) MPTP: F_(1, 28)_=4.565, *P*=0.042, NE100 F_(1, 28)_=2.624, *P*=0.116, MPTP × NE100: F_(1, 28)_=3.110, *P*=0.089; *P<0.05 *versus* WT mice and ^#^*P*<0.05 *versus* MPTP-WT mice (Fisher's LSD); (**e**) MPTP: F_(1, 28)_=7.378, *P*=0.011, PRE084: F_(1, 28)_=2.098, *P*=0.159, MPTP × PRE084: F_(1, 28)_=2.473, *P*=0.127; *P<0.05 *versus* MPTP-*σ*_1_R^+/−^ mice (Fisher's LSD)

The MPTP-induced death of dopaminergic neurons is known to produce motor deficits. Tyrosine hydroxylase (TH)-positive cells in SNpc were examined after the behavioral examination (Figure 1). The stereological counts showed that the number of TH-positive cells in *σ*_1_R^−/−^ mice did not differ from WT mice (*P*>0.05, *n*=8; Figure 4a). In comparison with WT mice, the number of TH-positive cells was reduced ~40% in MPTP-WT mice (*P*<0.05, *n*=8) but only 5% in MPTP-*σ*_1_R^−/−^ mice (*P*>0.05, *n*=8). Similarly, MPTP-WT mice showed an obvious decrease in the TH-positive fibers of dorsolateral striatum, but not MPTP-*σ*_1_R^−/−^ mice, relative to controls (*n*=8; Figure 4b). As shown in Figure 4c, a large number of Hoechst-positive cells in SNpc was observed in MPTP-WT mice, whereas hardly shown in MPTP-*σ*_1_R^−/−^ mice. The treatment with NE100 in MPTP-WT mice could attenuate the loss of TH-positive cells (*P*<0.05, *n*=8; Figure 4d). In addition, the number of TH-positive cells in MPTP-*σ*_1_R^+/−^ mice did not differ significantly from *σ*_1_R^+/−^ mice (*P*>0.05, *n*=8; Figure 4e). The administration of PRE084 caused 30% loss of TH-positive cells in MPTP-*σ*_1_R^+/−^ mice (*P*<0.05, *n*=8).

### *σ*_1_R deficiency reduces MPTP neurotoxicity via downregulation of NMDAr

**Figure 5:**
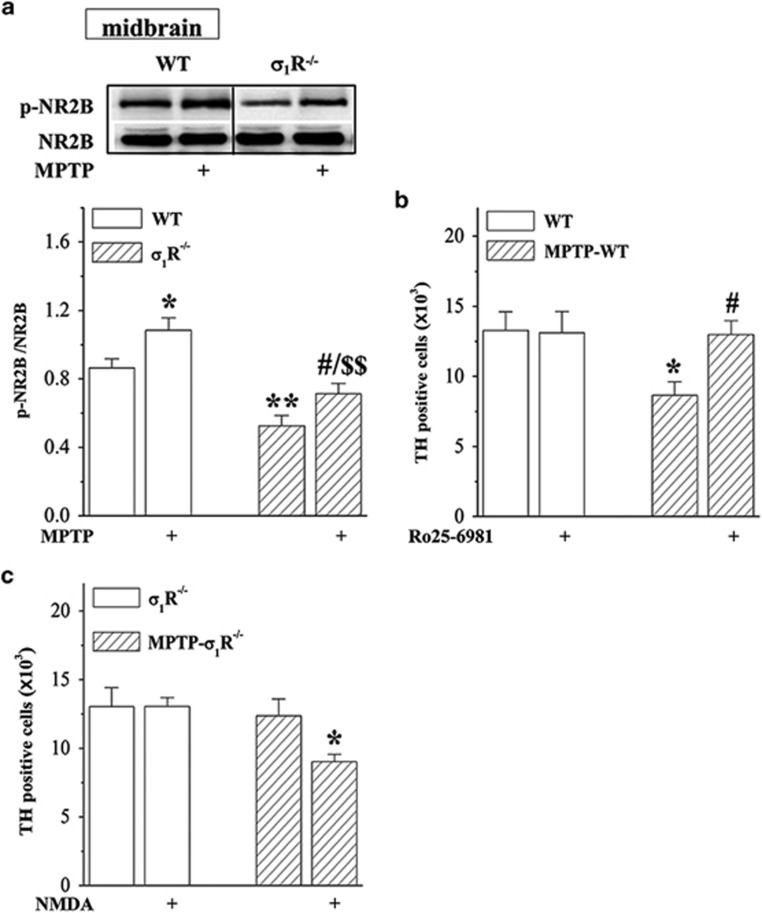
*σ*_1_R deficiency reduces MPTP-induced neurotoxicity via downregulation of NMDAr. (**a**) Levels of phospho-NR2B protein in midbrain area containing substantia nigra in WT mice and *σ*_1_R^−/−^ mice treated with MPTP injection. (**b**) Effects of NR2B inhibitor Ro25-6981 on MPTP-induced death of dopaminergic neurons. (**c**) Influence of NMDAr agonist NMDA on dopaminergic neurons in *σ*_1_R^−/−^ mice and MPTP-*σ*_1_R^−/−^ mice. Bar graphs show stereological counts of TH-positive cells. Two-way ANOVA, (**a**) MPTP: F_(1, 28)_ =13.007, *P*=0.001; genotype: F_(1, 28)_=29.096, *P*<0.001; MPTP × genotype: F_(1, 28)_=0.365, *P*=0.551; *P<0.05 and **P<0.01 *versus* WT mice, ^#^*P*<0.05 *versus σ*_1_R^−/−^ mice, ^$$^*P*<0.01 *versus* MPTP-WT mice (Fisher's LSD); (**b**) MPTP: F_(1, 28)_=3.930, *P*=0.057; Ro25-6981: F_(1, 28)_=2.501, *P*=0.125; MPTP × Ro25-6981: F_(1, 28)_=2.974, *P*=0.096; *P<0.05 *versus* WT mice, ^#^*P*<0.05 *versus* MPTP-WT mice (Fisher's LSD); (**c**) MPTP: F_(1, 28)_=5.468, *P*=0.027; NMDA: F_(1, 28)_=2.718, *P*=0.110; MPTP × NMDA: F_(1, 28)_=2.772, *P*=0.107; *P<0.05 *versus* MPTP-*σ*_1_R^−/−^ mice (Fisher's LSD)

To test the involvement of NMDAr in the *σ*_1_R deficiency-reduced MPTP neurotoxicity, we examined the level of NR2B phosphorylation (phospho-NR2B) in midbrain containing substantia nigra. Densitometry analysis revealed that the level of phospho-NR2B in *σ*_1_R^−/−^ mice was lower than that in WT mice (*P*<0.01, *n*=8; Figure 5a). Although the treatment with MPTP for 2 weeks (Figure 1) could increase the level of phospho-NR2B in WT mice (*P*<0.05, *n*=8) and MPTP-*σ*_1_R^−/−^ mice (*P*<0.05, *n*=8) without the change in the level of NR2B protein (*P*>0.05, *n*=8), the level of phospho-NR2B in MPTP-*σ*_1_R^−/−^ mice was still lower than that in MPTP-WT mice (*P*<0.01, *n*=8). In particular, the administration of NR2B inhibitor Ro25-6981 (6 mg/kg) in period of the MPTP injection (Figure 1) could reduce the death of TH-positive cells in MPTP-WT mice (*P*<0.05, *n*=8; Figure 5b). The treatment with the NMDAr agonist NMDA (30 mg/kg) led to the loss of TH-positive cells in MPTP-*σ*_1_R^−/−^ mice (*P*<0.05, *n*=8; Figure 5c), but not in *σ*_1_R^−/−^ mice (*P*>0.05, *n*=8).

### *σ*_1_R deficiency reduces DAT expression in dopaminergic neurons

**Figure 6:**
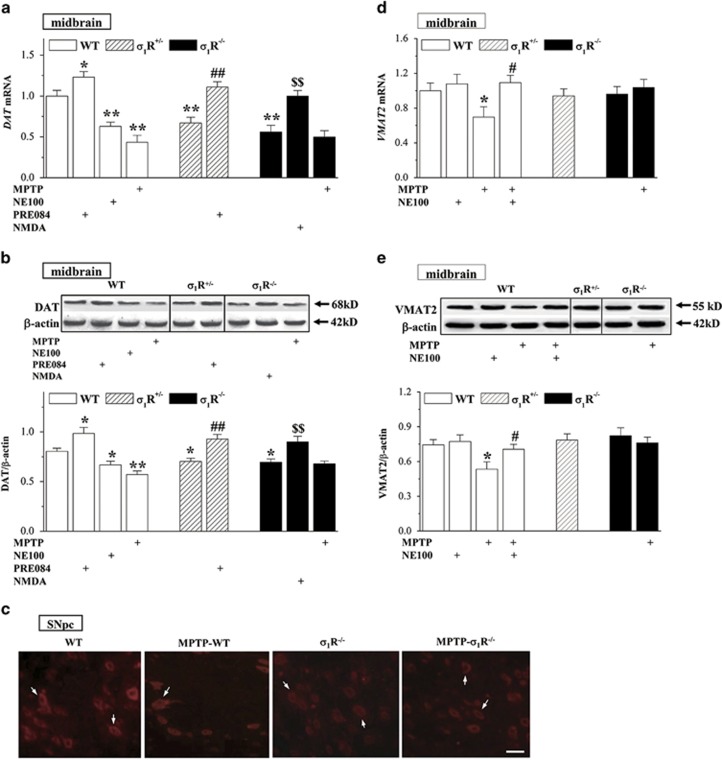
*σ*_1_R deficiency reduces DAT expression. (**a** and **b**) Levels of *DAT* mRNA and DAT protein in midbrain containing substantia nigra. Two-way ANOVA, (**a**) treatment (drugs and MPTP): F_(4, 63)_=28.325, *P*<0.001; genotype: F_(2, 63)_=4.942, *P*=0.010; treatment × genotype: F_(2, 63)_=6.374, *P*=0.003; *P<0.05 and **P<0.01 *versus* WT mice, ^##^*P*<0.01 *versus σ*_1_R^+/−^ mice, ^$$^*P*<0.01 *versus σ*_1_R^−/−^ mice (Fisher's LSD); (**b**) treatment: F_(4, 63)_=20.701, *P*<0.001; genotype: F_(2, 63)_=0.880, *P*=0.420; treatment × genotype: F_(2, 63)_=3.495, *P*=0.036; *P<0.05 and **P<0.01 *versus* WT mice, ^##^*P*<0.01 *versus σ*_1_R^+/−^ mice, ^$$^*P*<0.01 *versus σ*_1_R^−/−^ mice (Fisher's LSD). (**c**) Representative images of DAT immunostaining in SNpc. Scale bar=50 *μ*m. White arrows indicate DAT-positive dopaminergic neurons. (**d** and **e**) Levels of *VAMT2* mRNA and VAMT2 protein in midbrain containing substantia nigra. Three-way ANOVA, (**d**) MPTP: F_(1, 49)_=0.737, *P*=0.395; NE100: F_(1, 49)_=3.204, *P*=0.080; genotype: F_(2, 49)_=2.044, *P*=0.140; *P<0.05 *versus* WT mice, ^#^*P*<0.05 *versus* MPTP-WT mice (Fisher's LSD); (**e**) MPTP: F_(1, 49)_=2.253, *P*=0.140; NE100: F_(1, 49)_=3.411, *P*=0.071; genotype: F_(2, 49)_=2.184, *P*=0.123; *P<0.05 *versus* WT mice, ^#^*P*<0.05 *versus* MPTP-WT mice (Fisher's LSD)

To investigate the mechanisms underlying the *σ*_1_R deficiency-reduced MPTP neurotoxicity, we further analyzed the levels of DAT and vesicular monoamine transporter 2 (VMAT2) in midbrain containing substantia nigra. In comparison with WT mice, the levels of *DAT* mRNA (Figure 6a) and DAT protein (Figure 6b) were reduced in *σ*_1_R^+/−^ mice and *σ*_1_R^−/−^ mice or in NE100-treated WT mice (*DAT* mRNA: *P*<0.01, *n*=8; DAT protein: *P*<0.05, *n*=8), whereas levels were increased in PRE084-treated WT mice (*P*<0.05, *n*=8). The treatment of *σ*_1_R^+/−^ mice with PRE084 or the administration of NMDA in *σ*_1_R^−/−^ mice could correct the reduction in *DAT* mRNA (*P*<0.01, *n*=8) and DAT protein (*P*<0.01, *n*=8). In addition, MPTP-WT mice showed an obvious decline in the levels of *DAT* mRNA (*P*<0.01, *n*=8) and DAT protein (*P*<0.01, *n*=8) compared with WT mice, but MPTP-*σ*_1_R^−/−^ mice did not (*P*>0.05 *versus σ*_1_R^−/−^ mice, *n*=8). The immunofluorescence for DAT showed the localization of this protein in the bodies of dopaminergic neurons (Figure 6c). The immunointensity of DAT-positive cells in *σ*_1_R^−/−^ mice and MPTP-*σ*_1_R^−/−^ mice was weaker than that in WT mice. Although the number of DAT-positive cells was significantly reduced in MPTP-WT mice compared with WT mice, their immunointensity remained unaffected. By contrast, the levels of *VMAT2* mRNA (Figure 6d) and VMAT2 protein (Figure 6e) in *σ*_1_R^+/−^ mice or *σ*_1_R^−/−^ mice had no significant difference from WT mice (*P*>0.05, *n*=8). The treatment of WT mice with NE100 failed to alter the levels of *VMAT2* mRNA (*P*>0.05, *n*=8) and VMAT2 protein (*P*>0.05, *n*=8). The levels of *VMAT2* mRNA and VMAT2 protein were reduced in MPTP-WT mice (*P*<0.05, *n*=8), but not in MPTP-*σ*_1_R^−/−^ mice (*P*>0.05, *n*=8). Moreover, the decline of VMAT2 levels in MPTP-WT mice could be rescued by NE100 (*P*<0.05, *n*=8).

### *σ*_1_R deficiency reduces MPTP-induced astrocyte activation

**Figure 7:**
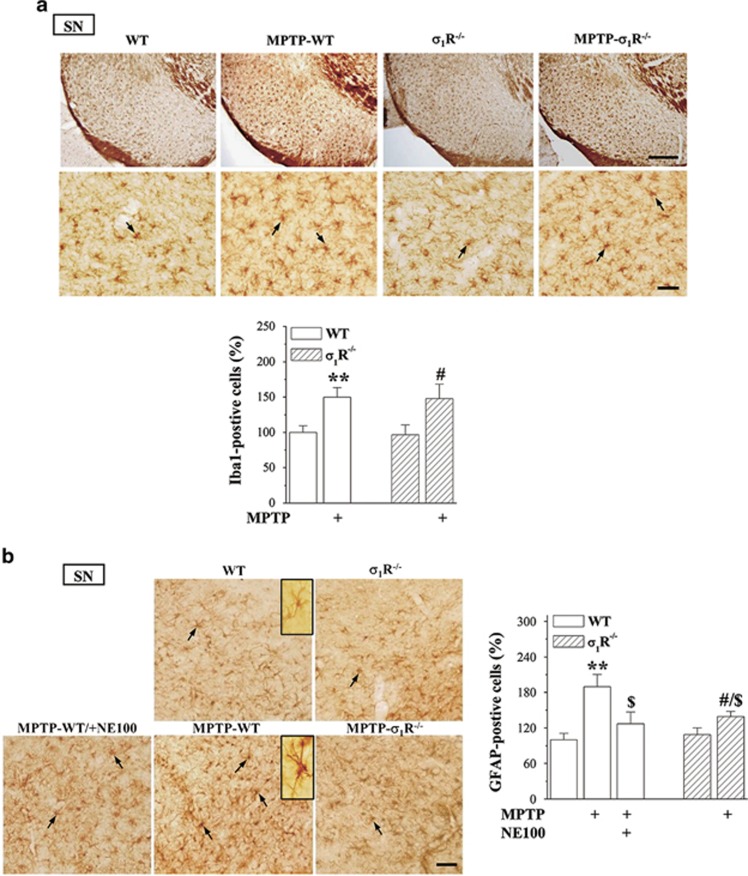
*σ*_1_R deficiency reduces MPTP-induced astrocyte activation. (**a**) Representative photomicrographs showing Iba1-positive microglial cells (black arrows) in substantia nigra (upper panels: scale bar=200 *μ*m; bottom panels: scale bar=50 *μ*m). Bar graph shows number of Iba1-positive cells in *σ*_1_R^−/−^ mice and MPTP-*σ*_1_R^−/−^ mice that are normalized by the number of Iba1-positive cells obtained in WT mice. (**b**) Representative photomicrographs showing GFAP-positive cells (black arrows) in substantia nigra. Scale bar=50 *μ*m. GFAP-positive cell (upper inset) in WT mice represents a resting astrocyte. GFAP-positive cell (bottom inset) in MPTP-WT mice reflects activation state of astrocyte. Two-way ANOVA, (**a**) MPTP: F_(1, 28)_=26.758, *P*<0.001; genotype: F_(1, 28)_=1.332, *P*=0.258; MPTP × genotype: F_(1, 28)_=0.164, *P*=0.689; **P<0.01 *versus* WT mice; ^#^*P*<0.05 *versus σ*_1_R^−/−^ mice (Fisher's LSD); (**b**) treatment (NE100 and MPTP): F_(2, 35)_=7.865, *P*=0.002; genotype: F_(1, 35)_=2.376, *P*=0.132, treatment × genotype: F_(1, 35)_=3.202, *P*=0.082; **P<0.01 *versus* WT mice; ^#^*P*<0.05 *versus σ*_1_R^−/−^ mice; ^$^*P*<0.05 *versus* MPTP-WT mice (Fisher's LSD)

The Iba1-positive microglia cells and glial fibrillary acidic protein (GFAP)-positive astrocytes were examined in substantia nigra (Figure 7a). The number of Iba1-positive cells had no significant difference between WT mice and *σ*_1_R^−/−^ mice (*P*>0.05, *n*=8). MPTP-WT mice or MPTP-*σ*_1_R^−/−^ mice showed an obvious increase in the number of Iba1-positive cells compared with WT mice (*P*<0.01, *n*=8) or *σ*_1_R^−/−^ mice (*P*<0.05, *n*=8), but the group comparison failed to show difference between MPTP-WT mice and MPTP-*σ*_1_R^−/−^ mice (*P*>0.05, *n*=8). The morphological analysis (Figure 7b) showed GFAP-positive stellated-shaped astrocytes with thin processes denoting resting phenotype, and stronger GFAP immunoreactivity astrocytes with thick processes reflecting activated phenotype.^19^ The number of activated GFAP-positive cells in *σ*_1_R^−/−^ mice did not differ significantly from WT mice (*P*>0.05, *n*=8). In comparison with WT mice, the number of activated GFAP-positive cells was increased approximately 2-fold in MPTP-WT mice (*P*<0.01, *n*=8), but only 1.3-fold in MPTP-*σ*_1_R^−/−^ mice (*P*<0.05, *n*=8). Thus, the number of activated GFAP-positive cells in MPTP-*σ*_1_R^−/−^ mice was less than that in MPTP-WT mice (*P*<0.05, *n*=8). Moreover, the number of activated GFAP-positive cells in MPTP-WT mice could be reduced by the administration of NE100 (*P*<0.05, *n*=8).

## Discussion

Using the MPTP-*σ*_1_R^+/−^ and MPTP-*σ*_1_R^−/−^ mice models, the present study provides evidence that the *σ*_1_R deficiency can reduce the MPTP-induced parkinsonism and death of dopaminergic neurons. This conclusion is deduced mainly from the following results. The MPTP-induced motor deficits and death of dopaminergic neurons in WT mice were alleviated by the blockade of *σ*_1_R. By contrast, MPTP failed to induce the motor deficits and the death of dopaminergic neurons in *σ*_1_R^+/−^ mice and *σ*_1_R^−/−^ mice. MPTP-*σ*_1_R^+/−^ mice treated with the *σ*_1_R agonist PRE084 (1.0 mg/kg) or MPTP-*σ*_1_R^−/−^ mice treated with NMDA appeared to have the same motor deficits and death of dopaminergic neurons as MPTP-WT mice. There is, however, an apparently conflicting report describing that the administration of PRE084 at the dose of 0.3 mg/kg, but not the dose of 1.0 mg/kg, can improve the spontaneous forelimb use and reduce the death of dopaminergic neurons in mice subjected to 6-OHDA lesion.^4^ The behavioral, biochemical or electrophysiological effects of *σ*_1_R agonists show a biphasic, inverted U (bell) shape and dose-dependent.^20^ Thus, this discord may arise from the difference in the experimental models or the dose of PRE084 used.

### Influence of *σ*_1_R deficiency-reduced NMDAr activation on motor behaviors

The first question we should address may be whether the *σ*_1_R deficiency affects the motor behaviors. Using OFT and BWT, we found that pharmacological or genetic inactivation of *σ*_1_R failed to affect the locomotion ability and motor coordination, which are in agreement with previous studies.^21, 22^ In addition, *σ*_1_R^−/−^ mice did not appear the spontaneous turning behavior and forelimb use asymmetry as assessed by the cylinder test and the stepping test.^4^ The stride length in the footprint test that is a sensitive assay for motor coordination shows no difference between WT mice and *σ*_1_R^−/−^ mice and the swimming speed in the swimming test in *σ*_1_R^−/−^ mice is faster than controls.^3^ The time stayed on the rotated rod with constant speeds (4–36 r.p.m.) in *σ*_1_R^+/−^ and *σ*_1_R^−/−^ mice had no significant difference from WT mice. There are, however, conflicting reports describing the motor coordination defects in *σ*_1_R^−/−^ mice that is detected by accelerating RT with a rate of 4–50 r.p.m. for 2.5 min.^3, 23^ The contradictory results may arise from the difference in the protocol of RT, because we observed that *σ*_1_R^−/−^ mice, but not *σ*_1_R^+/−^ mice, showed a short latency on the accelerating rotarod in comparison with WT mice (data not shown). Monville *et al.* reported that the incremental constant protocol in RT is more sensitive to detect the presence of lesion, whereas the accelerating protocol provides a more discriminative test to correlate motor deficits.^24^ Rustay *et al.* found that both accelerating and fixed-speed rotarod performance can vary under different condition.^25^ On the other hand, the NMDAr is essential for mediating excitatory transmission at corticostriatal dopaminergic synapses. The activation of *σ*_1_R increases both dopamine and glutamate release.^7, 26^ In addition to degeneration of dopaminergic neurons, abnormal function of striatal NMDAr has been implicated in the development of motor deficits.^27^ Recently, Xiu *et al.* have reported that the chronic treatment with the NMDAr antagonist MK801 does not affect the coordination or motor function on accelerating rotarod.^28^ Thus, it is conceivable that the *σ*_1_R deficiency-reduced NMDAr function does not produce the motor deficits.

### *σ*_1_R deficiency suppresses NMDAr to prevent MPTP neurotoxicity

Glutamate toxicity has been noted as a main source of the MPTP-impaired dopaminergic system.^29^ The tyrosine phosphorylation of NR2B is enhanced by the application of 6-OHDA.^30^ The blockade or the knockout of *σ*_1_R can reduce the levels of phospho-NR2B in substantia nigra or hippocampus.^31^ More importantly, the MPTP-induced increase of phospho-NR2B was attenuated by the blockade or the knockout of *σ*_1_R. One earlier study has reported the MPTP binding to *σ* protein in C57BL/6 mouse brain membranes.^32^ Thus, further studies are needed to evaluate whether MPTP via the activation of *σ*_1_R enhances the phospho-NR2B. The blockade of NMDAr can reduce the death of dopaminergic neurons in monkey and mouse PD models.^33, 34^ Consistent with an earlier study,^12^ the NMDAr NR2B inhibitor could prevent the MPTP-induced death of dopaminergic neurons. The NMDAr agonist NMDA increased the loss of dopaminergic neurons in MPTP-*σ*_1_R^−/−^ mice. Therefore, one possible explanation is that the *σ*_1_R deficiency through suppressed MPTP-induced increase of phospho-NR2B can prevent NMDAr-mediated death of dopaminergic neurons.

### *σ*_1_R deficiency reduces DAT to prevent MPTP neurotoxicity

An important finding in this study is that the pharmacological or genetic inactivation of *σ*_1_R reduced the DAT expression in dopaminergic neurons, but failed to affect the VMAT2 level. The compensatory downregulation of DAT in early PD is thought to maintain dopamine levels in the synapse.^35^ Zhang and Li have recently reported that DAT expression depends on the striatal extracellular dopamine concentration.^36^ The activation of *σ*_1_R through enhancing NMDAr can increase the dopamine release.^7^ Indeed, the application of NMDA in *σ*_1_R^−/−^ mice could enhance the expression of DAT. Therefore, one possible explanation is that the *σ*_1_R deficiency reduces the release of dopamine via the suppression of NMDAr leading to the compensatory decline in the expression of DAT. On the other hand, MPTP-WT mice appeared the reduction of DAT and VMAT2 levels, which could be rescued by the blockade or knockout of *σ*_1_R. An earlier study reported that the MPTP-induced loss of dopaminergic neurons was accompanied by a decline in DAT and VMAT2 levels.^37^ Therefore, it is suggested that the reduction of DAT and VMAT2 in MPTP-WT mice arises from the loss of dopaminergic neurons. *In vivo* studies have shown that the knockout or the inhibition of DAT can prevent MPTP-induced neurotoxicity.^38, 39^ The DAT-overexpressing mice are highly sensitive to MPTP neurotoxicity.^9^ The increased dopamine uptake is able to elevate the unique vulnerability of dopamine neurons in PD. Thus, it is possible that the *σ*_1_R deficiency through downregulating DAT expression can reduce the MPTP-impaired dopaminergic neurons.

### *σ*_1_R deficiency suppresses MPTP-induced astrocyte activation

Consistent with an earlier study in the mouse model,^40^ the injection of MPTP could stimulate the activation of astrocytes or microglia cells. In mice of intrastriatal 6-OHDA lesions, the microglia cell activation contributes to the neurodegenerative process during the rapid phase of dopamine cell death, and persists for weeks.^41^ The *σ*_1_R is expressed in astrocytes and microglia cells.^42^ The 6-OHDA-induced microglia cell activation can be attenuated by the *σ*_1_R agonist PRE084.^4^ The activation of *σ*_1_R has the anti-inflammatory effects in ALS mice.^43^ However, our results showed that the *σ*_1_R deficiency had no effect on MPTP-induced activation of microglia cells. By contrast, the number of activated astrocyte in MPTP-*σ*_1_R^−/−^ mice was less than that in MPTP-WT mice. In addition, the blockade of *σ*_1_R by NE100 in MPTP-WT mice could reduce the activated astrocyte. Recently, a *σ*_1_R antagonist has been reported to attenuate methamphetamine-induced neurotoxicity and astrogliosis through a blockade of oncostatin M receptor/gp130 signaling and STAT3 phosphorylation.^16, 44^ Therefore, the reduced astrocyte activation in MPTP-*σ*_1_R^−/−^ mice is thought to have two possibilities: one is that the *σ*_1_R deficiency reduces the MPTP-induced neuronal damage and the other is that the *σ*_1_R deficiency can suppress the astrocyte activation. Further work will be required to determine these possibilities.

The decreased *σ*_1_R-binding site in early Alzheimer's disease is reported to have a protective effect against Alzheimer's disease susceptibility in a Japanese population.^45^ By contrast, the high expression of *σ*_1_R with the APOE *ɛ*4 allele in Chinese or Australian populations has been reported to advance cognitive dysfunction and pathologic stages of Alzheimer's disease.^46^ Despite the association between the reduction of *σ*_1_R and the early PD has not been reported, the present study suggests that the *σ*_1_R deficiency in the mice model of MPTP-induced PD might exert the neuroprotection effects, which would open new doors for preventing and treating PD.

## Materials and Methods

### Animals

All animal handling procedures followed the Guidelines for Laboratory Animal Research of Nanjing Medical University. The use of animals was approved by the Institutional Animal Care and Use Committee of Nanjing Medical University. As a *σ*_1_R knockout line, we chose the well-characterized Oprs1 mutant (+/−) Oprs1Gt(IRESBetageo)33Lex litters on a C57BL/6 J × 129 S/SvEv mixed background,^21^ which we obtained from the Mutant Mouse Resource Regional Centre at the University of California, Davis. The genotype of mice was identified by PCR using genomic DNA from tail biopsies (Supplementary Figure 1A) with the following primers sequences: (a) 5′-TCTGAGTACGTGCTGCTCTTCG-3′ (b) 5′-ATAAACCCTCTTGCAGTTGCATC-3′ (c) 5′-GAAACTGCCGTGTTCTGTTTCC-3′, and PCR reaction parameters: 30 cycles of 94 °C (15 s), 55 °C (30 s) and 72 °C (40 s).^21^ The western blotting analysis showed the reduction or lack of *σ*_1_R protein level in *σ*_1_R^+/−^ mice and *σ*_1_R^−/−^ mice, respectively (Supplementary Figure 1B). The mice were maintained under constant environmental conditions (temperature 23±2 °C, humidity 55±5% and 12 : 12- h light/dark cycle) in the Animal Research Center of Nanjing Medical University with free access to food and water. Consistent with the report by Sabino *et al.*,^21^ the development of *σ*_1_R^+/−^ mice and *σ*_1_R^−/−^ mice appeared grossly normal.

### Experimental design

Male 12-week-old WT mice (25.26±0.84 g), *σ*_1_R^+/−^ mice (25.19±0.91 g) and *σ*_1_R^−/−^ mice (25.35±0.76 g) were used at the beginning of all experiments. A total of 176 WT mice, 80 *σ*_1_R^+/−^ mice and 120 *σ*_1_R^−/−^ mice were divided into five experimental groups to examine (a) influence of *σ*_1_R deficiency on MPTP-impaired motor behaviors (*n*=12) and MPTP neurotoxicity (*n*=8); (b) effects of *σ*_1_R antagonist and agonist on MPTP-impaired motor behaviors (*n*=12) and MPTP neurotoxicity (*n*=8); (b) influence of *σ*_1_R deficiency on MPTP-increased NR2B phosphorylation (*n*=8) and NMDAr-mediated MPTP neurotoxicity (*n*=8); (d–e) influence of *σ*_1_R deficiency on DAT and VAMT2 expression (*n*=8) and MPTP-induced inflammatory (*n*=8). Behavioral tests were carried out starting from day 3 after the last injection of MPTP or drugs to evaluate spontaneous locomotion and motor coordination. At the end of the behavioral tests, the mice were perfusion-fixed for histological examination (time chart of experimental procedure; Figure 1). In addition, on day 3 after the last injection of MPTP, the mice were decapitated for either biochemical assays or western blotting.

### Treatment with drugs

MPTP and probenecid (Sigma-Aldrich, St. Louis, MO, USA) were dissolved in 0.9% sterile saline and dimethylsulfoxide, respectively. The mice received a subcutaneous (s.c.) injection of MPTP (25 mg/kg) and an intraperitoneal (i.p.) injection of probenecid (250 mg/kg) for 10 times with intervals of 3.5 days (Figure 1).^47^ N-methyl-D-aspartic acid (NMDA), R-(R*, S*)-α-(4-hydroxyphenyl)-beta-methyl-4-(phenyl methyl)-1-piperidinepropanol (Ro25-6981) and 2-(4-morpholineylethyl) 1-phenylcyclohexane-1-carboxylate (PRE084) were purchased from Sigma-Aldrich and dissolved in 0.9% sterile saline. N,N-dipropyl-2-[4-methoxy-3-(2-phenylethoxy) phenyl] ethylamine hydrochloride (NE100) was kindly supplied by Taisho Pharmaceutical Co. Ltd (Tokyo, Japan) and was dissolved in distilled water. Ro25-6981 (6 mg/kg), NMDA (30 mg/kg), PRE084 (1 mg/kg) and NE100 (1 mg/kg) were daily administered (i.p.)^13^ for 5 weeks. Control mice were given an equal volume of vehicle.

### Behavioral examination

Three different behavioral tests were carried out (9000-1400) under following sequence: OFT→BWT→RT. Tests were spaced by 24 h. These behavioral tests were recorded by a video monitor (Winfast PVR; Leadtek Research Inc., Fremont, CA, USA). The results of OFT and BWT were analyzed using TopScan Lite 2.0 (Clever Sys, Reston, VA, USA) and the results of RT were analyzed by Rota-Rod microprocessor 47600 (Ugo Basile, Biological Research Apparatus, Varese, Italy).

#### Open-field test

Each mouse was placed in a clear, open-top, square Plexiglas box (30 × 30 × 40 cm^3^) in a subdued room and allowed to freely explore for 6 min. Rearing number and traveled distance were measured within 6 min.^48^

#### Beam walking test

The challenging beam was a 1-m long wooden beam suspended 23 cm above a bench top, which was covered with soft pads to protect the mouse in case of a fall. The beam was divided in four gradually narrowing sections (25 cm/section) leading to the mouse's home cage. The beam widths of the four sections were 3.5, 2.5, 1.5 and 0.5 cm in decreasing order. The beam was covered with surgical tape that provided sufficient surface traction for the animals to walk on. There were 1-cm-wide ledges hanging 1 cm below each side of the beam to encourage the mice to use their normal gait strategies even when their limbs slipped. All mice were pre-trained for 2 consecutive days on traversing the beam. On the third day, each mouse was given five trials (inter-trial intervals=10–12 s), and the average time was calculated.^49^

#### Rotarod test

The rotarod apparatus (Ugo Basile) was used to measure forelimb and hindlimb motor coordination and balance. Two different protocols are widely used, incremental fixed speeds or an accelerating protocol. The former may be most appropriate to detect the presence of a lesion, whereas the latter is to be preferred to characterize the magnitude and extent of depletion in different animals.^24^ The first protocol was selected in this study to examine MPTP-induced parkinsonism. Mice were placed on the rotating rod using the following steps: on days 1 and 2, mice learned to stay on the rotarod at constant speed (20 r.p.m.) for 300 s. At day 3, motor coordination was assessed on the rotarod with five constant speeds (4–36 r.p.m.) for a maximum of 60 s at each speed. For each trial, the time until the mice fall off the rod was recorded. The animals were tested two times at each speed with a rest of 20 min between each trial.^24^

### Histological examination and quantitative analyses

Mice were anesthetized with chloral hydrate (400 mg/kg, i.p.), and then perfused with 4% paraformaldehyde. Coronal sections (30 *μ*m) were cut using a cryostat. The immunostaining of TH, glial fibrillary acidic protein (GFAP) and ionized calcium-binding adapter molecule 1 (Iba1) was performed using the following primary antibodies: chicken anti-TH (1 : 1000; Abcam, Cambridge, UK), rabbit anti-GFAP (1 : 1000; Cell Signaling Technology, Inc., Boston, MA, USA) and goat anti-Iba1 (1 : 1000; Abcam) at 4 °C overnight. Then the sections were incubated in biotin-labeled goat anti-chicken IgG antibody (1 : 500; Santa Cruz Biotechnology, Santa Cruz, CA, USA), goat anti-rabbit IgG antibody (1 : 400; Santa Cruz) or rabbit anti-goat antibody (1 : 400; Santa Cruz). Immunoreactivity was visualized by the avidin–biotin–horseradish peroxidase complex (ABC Elite; Vector Laboratories, Inc., Burlingame, CA, USA). For DAT immunostaining, the brains were processed for paraffin embedding. Coronal paraffin sections (5 *μ*m) were incubated in rabbit anti-DAT antibody (1 : 200; Alomone Labs, Jerusalem, Israel) at 4 °C overnight. The sections were incubated in Cy3-conjugated goat anti-rabbit antibody (1 : 200, Jackson ImmumoResearch Lab., West Grove, PA, USA) for 2 h. For Hoechst staining, the paraffin sections (5 μm) were incubated in Hoechst 33342 (1 *μ*g/ml; Cell Signaling Technology) for 2 min. The DAT-positive cells and Hoechst-positive cells were observed using a fluorescent light microscope (Olympus, DP70; Tokyo, Japan).

The number of TH-positive cell bodies in SNpc (12 sections per mouse) was determined by Microbrightfield Stereo Investigator software according to the optical fractionator method (Microbrightfield, Williston, VT, USA).^50^ The total number of TH-positive cells was estimated using the optical fractionator formula: number of neurons=1/ssf (slice sampling fraction) × 1/asf (area sampling fraction) × 1/tsf (thickness sampling fraction) × Σ (number of objects counted). The Iba1- and GFAP-positive cells in substantia nigra (12 sections per mouse) were counted in an area of 500 × 500 *μ*m^2^ per section by a conventional light microscope (Olympus, DP70, × 40) with an *x*–*y* motorized stage controlled by the New CAST software (Visiopharm, Hørsholm, Denmark). The number of Iba1- and GFAP-positive cells was normalized by value of WT mice.

### Western blotting analysis

Mice were anesthetized with chloral hydrate. The midbrain containing substantia nigra was taken quickly and homogenized in a lysis buffer containing 50 mM Tris-HCl (pH 7.5), 150 mM NaCl, 5 mM EDTA, 10 mM NaF, 1 mM sodium orthovanadate, 1% Triton X-100, 0.5% sodium deoxycholate, 1 mM phenyl-methylsulfonyl fluoride and a protease inhibitor cocktail (Complete; Roche, Mannheim, Germany). Total proteins (20 μg) were separated by SDS-PAGE and transferred to a polyphorylated difluoride membrane. The membranes were incubated with the antibody of rabbit anti-NR2B phosphorylation (1 : 1000; Abcam), rabbit anti-DAT (1 : 200; Alomone Labs), rabbit anti-VMAT2 (1 : 500; Millipore, Billerican, MA, USA) and rabbit anti-*σ*_1_R (1 : 500; Santa Cruz) at 4 °C overnight. After appropriate washing steps, the membranes were incubated with horseradish peroxidase-linked goat anti-rabbit antibody (1 : 10 000; Millipore). Following visualization, the blots were stripped by incubation in stripping buffer (Restore; Pierce Biotechnology, Inc., Rockford, IL, USA) for 15 min and then incubated with antibodies of rabbit anti-NR2B (1 : 1000; Millipore) or rabbit anti-*β*-actin (1 : 1000; Abcam). Western blot bands were scanned and analyzed with the image analysis software package (Image J; NIH Image, Bethesda, MD, USA).

### Quantitative real-time reverses transcription-PCR (Q-RT-PCR)

Midbrain containing substantia nigra was taken quickly, and total RNA was isolated using TRIzol reagent (Invitrogen, Carlsbad, CA, USA). RNA (1 μg) was used to reverse transcribe using high-capacity cDNA of the reverse transcription kit RT (TaKaRa Biotechnology CO., Ltd, Dalian, China). The primer sequences of *DAT* (forward primer: 5′-ATCAACCCACCGCAGACACCAGT-3′ reverse primer: 5′-GGCATCCCGGCAATAACCAT-3′) and *VAMT2* mRNA (forward primer: 5′-ATGCTATCGGTCCCTCTGCTGGTG-3′; reverse primer: 5′-GACGGGGTACGGCTGGACATTATT-3′) were designed according to a previous publication.^51^ Q-RT-PCR was performed using a Light Cycler Fast Start DNA Master SYBR Green I kit and an ABI Prism 7300 Sequence Detection System (Applied Biosystems, Foster City, CA, USA). The relative expression of genes was determined using the 2-ΔΔct method, with normalization to *GAPDH* expression. The levels of *DAT* and *VAMT2* mRNA were expressed as percent of WT mice.

### Data analysis/statistics

Data were retrieved and processed with Micro cal Origin 6.1 (Origin Lab, Northampton, MA, USA). The group data were expressed as the means±standard error (S.E.). All statistical analyses were performed using SPSS software, version 16.0 (SPSS Inc., Chicago, IL, USA). Differences among means were analyzed using the two/three-factor analysis of variance (ANOVA) with or without repeated-measures, followed by *post hoc* Fisher's LSD test, where appropriate. Differences at *P*<0.05 were considered statistically significant. The F-values of ANOVAs are given in the figure legends, and *post hoc* pairwise comparisons are presented in the results section as being significant or non-significant.

## Abbreviations

6-OHDA: 6-hydroxydopamine
BWT: beam walking test
DAT: dopamine transporter
GFAP: glial fibrillary acidic protein
MPTP: 1-methyl-4-phenyl-1,2,3,6-tetrahydropyridine
NMDAr: N-methyl-D-aspartate receptors
OFT: open-field test
PD: Parkinson's disease
RT: rotarod test
SNpc: substantia nigra pars compacta
TH: tyrosine hydroxylase
*σ*_1_R: Sigma-1 receptor
VMAT2: vesicular monoamine transporter 2
WT: wild type
