# Supplementary material for: Sigma-1 receptor deficiency reduces MPTP-induced parkinsonism and death of dopaminergic neurons
Source: Cell Death Dis. 2015 Jul 23;6(7):e1832–. doi: 10.1038/cddis.2015.194 (PMC4650739; doi:10.1038/cddis.2015.194)

**Supplementary Material**

**Figure S1. Genotyping examination of heterozygous and homozygous σ_1_R knockout (σ_1_R^+/-^ and σ_1_R^-/-^) mice.** (A) Typical PCR blot for a litter from WT mice, σ_1_R^+/-^ mice and σ_1_R^-/-^ mice. (B) Western blot analysis of mesencephalic σ_1_R protein levels in WT mice, σ_1_R^+/-^ mice and σ_1_R^-/-^ mice. ***P*<0.01 *vs*. WT mice.


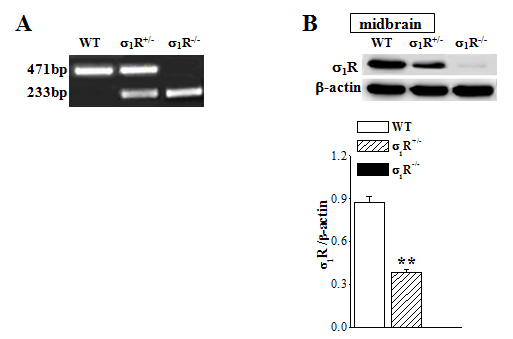

Supplement: Supplementary Material [file cddis2015194x1.doc]
